# Drone-Based Thermal Imaging in the Detection of Wildlife Carcasses and Disease Management

**DOI:** 10.1155/2023/5517000

**Published:** 2023-05-15

**Authors:** Janine Rietz, Suzanne T. S. van Beeck Calkoen, Nicolas Ferry, Jens Schlüter, Helena Wehner, Karl-Heinz Schindlatz, Tomáš Lackner, Christian von Hoermann, Franz J. Conraths, Jörg Müller, Marco Heurich

**Affiliations:** ^1^Department of National Park Monitoring and Animal Management, Bavarian Forest National Park, Freyunger Straβe 2, 94481 Grafenau, Germany; ^2^Chair of Wildlife Ecology and Management, Albert Ludwigs University Freiburg, Tennenbacher Straβe 4, 79106 Freiburg, Germany; ^3^Department of Conservation and Research, Bavarian Forest National Park, Freyunger Str. 2, 94481 Grafenau, Germany; ^4^Department of Animal Ecology and Tropical Biology, University of Würzburg, Würzburg, Germany; ^5^Friedrich-Loeffler-Institut, Institute of Epidemiology, 17493 Greifswald-Insel Riems, Germany; ^6^Faculty of Applied Ecology, Agricultural Sciences and Biotechnology, Inland Norway University of Applied Sciences, 2480 Koppang, Norway

## Abstract

Because animal carcasses often serve as reservoirs for pathogens, their location and removal are crucial in controlling the spread of diseases. During carcass decomposition, heat is emitted due to microbial activity and the development of maggots. Recent studies have shown that infrared sensors can be used to locate animal carcasses, but little is known about the factors influencing detection success. In this study, we investigated the potential of infrared technology to locate wild boar carcasses, as they play an important role in the spread of African swine fever. Specifically, we tested the effects of environmental and carcass conditions on the detection probability. A drone-based thermal camera was used to collect data during 379 flyovers of 42 wild boar carcasses in different stages of decomposition between September 2020 and July 2021. Generalized mixed-effect models and conditional inference trees were used to identify the environmental and carcass conditions that influenced the detection probability. Our results showed that the thermal camera accurately measured carcass temperature (*R*^2^ = 0.75, RMSE = 5.89°C). The probability of finding carcasses was higher in open habitats with air temperatures >3.0°C and thus conducive to maggot development (detection rate ≤80%). A forest canopy openness >29.3% and cloudy conditions or flights at dawn increased the detection rate. Moreover, carcasses infested with large amounts of maggots could be detected even in habitats with a more extensive canopy cover, whereas in dense forests, the detection probability was limited (<25%). Carcasses in an advanced stage of decomposition could still be detected as long as the difference between the carcass temperature and the air temperature was >6.4°C (≤62%). Our study demonstrates the utility of thermal imaging in searching for wild boar carcasses under specific environmental and carcass conditions and thus its use in supporting ground searches.

## 1. Introduction

In recent years, the increasing spread of infectious diseases by wildlife has raised concerns regarding the related threats to humans and domestic animals [1, 2]. Such rising pathogen emergence can be driven by growing anthropogenic impacts on nature, leading to increasing human-wildlife interactions [1, 3]. Contact between humans and wildlife can lead to the transmission of zoonotic diseases, while contact between wildlife and livestock threatens the health of domestic animals and, consequently, food production [4]. Developing strategies aimed at preventing the spread of diseases originating in wildlife is therefore of utmost importance. As animal carcasses play a key role in pathogen transmission [5], their timely removal is recommended to prevent disease spread, especially in the case of highly infectious diseases such as African swine fever [6, 7].

African swine fever (ASF) is a viral disease that originated in wild suids in Africa. However, it can be transmitted to other suids, including domestic pigs and wild boar (*Sus scrofa*), and has recently spread to Europe, Asia, and the Caribbean [8, 9], causing a pandemic among suids. The severity of the disease, as illustrated by the high case fatality ratio, and the transmission dynamics have led to massive economic losses in pig production and drastic reductions in the size of affected wild boar populations [7, 10]. Direct transmission of the ASF virus among wild boar can result from direct contact with infected animals or with the carcass of an infected wild boar (e.g., during scavenging or chewing on bones); indirect transmission is possible as well, such as by rooting in contaminated soil at the carcass site [6, 11, 12]. Because the carcasses of wild boar that have died from ASF can remain infectious for several months [13–15], they are considered to be one of the main drivers of ASF transmission [10, 16, 17].

To contain the disease, it is crucial that wild boar carcasses are found and removed quickly [15, 18, 19]. The active search for wild boar carcasses in high-risk areas has been proposed as the most efficient way to detect and counteract the disease [7, 20, 21]. However, the problem is that infected wild boars tend to hide and rest in more dense vegetation and choose those locations, especially for their deathbeds, which complicates subsequent discovery of their carcasses [22–24]. Developing and improving strategies for the rapid detection of wild boar carcasses is therefore crucial for a successful control of ASF [19, 20, 25].

Although a carcass cools down quickly after the death of the animal, its temperature rises again during decomposition. This is mainly due to the activities of necrophagous insects, whose closely packed larvae hatch within a short time period to form so-called maggot masses. Metabolic heat is generated by maggots feeding on body tissue, with the magnitude of the temperature rise increasing with increasing mass size [26]. Since maggot eggs are oviposited during the early stages of carcass decomposition, maggots often accelerate the decomposition process to a stage of active decay, during which the level of metabolic heat is highest [27–29]. Insect activity and the formation of maggot masses depend on the ambient temperature, as both are only possible above certain temperature thresholds [26, 30, 31]. The temperature characteristics and dependency of maggots can thus be exploited to improve carcass detection by thermal imaging [7].

Forward-looking infrared (FLIR) cameras (hereafter, thermal cameras) which can visualize heat-emitting objects have been used in the remote monitoring of wildlife [32], especially for the tracking of ungulates [33–37]. While thermal cameras were first mounted on helicopters [28, 29], with reductions in sensor weight they can now be used with unmanned aerial vehicles (hereafter, drones), which are relatively low cost and more flexible in their applications [27]. Temperature differences detected by thermal cameras have formed the basis of several forensic studies testing the feasibility of thermal imaging in the detection of human remains, with pig cadavers often used as human surrogates [27, 29, 38]. However, the application of thermal sensors is limited by several interfering factors, such as a dense forest canopy and the presence of other heat-emitting sources in the surrounding environment [39].

In this study, we examined the use of drone-based infrared imagery in finding wild boar carcasses, as part of the effort to contain diseases such as African swine fever. Specifically, we (1) tested the accuracy of a thermal camera in measuring carcass temperature and the biotic and abiotic factors that influence measurement accuracy. We then identified (2) the environment- and (3) carcass-related variables that determine the probability of carcass detection.

## 2. Methods

### 2.1. Study Area

Carcass exposure and drone flights were conducted in the Bavarian Forest National Park (BFNP, 249 km^2^, 49° 3′ 19″N, 13° 12′ 9″E), located in southeastern Germany. The elevation in the park ranges from 600 m.a.s.l to 1453 m.a.s.l on mountain peaks. The annual air temperature varies between 3.5 and 7.2°C, and the annual precipitation ranges from 830 mm to 1820 mm. Snow cover can persist from October to May, with the greatest depth from January to March [40, 41]. The area is covered by temperate forests composed mainly of Norway spruce (*Picea abies*), European beech (*Fagus sylvatica*), silver fir (*Abies alba*), common rowan (*Sorbus aucuparia*), and sycamore maple (*Acer pseudoplatanus*) [42]. The BFNP provides habitat to large mammals, including wild boar (*Sus scrofa*), red deer (*Cervus elaphus*), roe deer (*Capreolus capreolus*), and large carnivores, such as Eurasian lynx (*Lynx lynx*) and gray wolf (*Canis lupus*). Mesocarnivores, such as red fox (*Vulpes vulpes*) and badger (*Meles meles*), as well as different bird species, such as common buzzard (*Buteo buteo*) and raven (*Corvus corax*), are frequent scavengers in the study area.

### 2.2. Experimental Design

**Figure 1:**
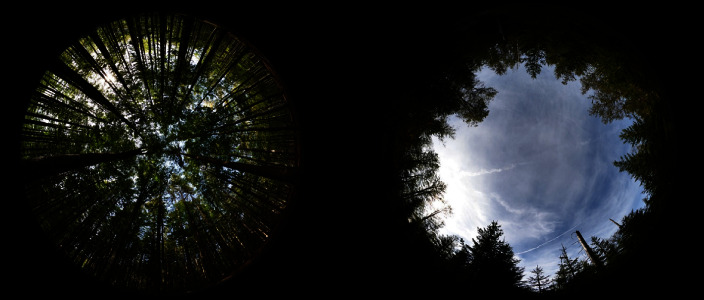
Two forests with different canopy openness. Percentage of canopy openness was used as a predictor variable to analyze its influence on the probability of finding carcasses using thermal imaging from drones.

Between September 2020 and July 2021, 42 wild boar carcasses were regularly deployed at a rate of four carcasses every 3 weeks. Due to high snow cover, cold temperatures, and thus the expected lack of visible carcass decomposition, deployment and drone flights were not performed between 23^rd^ November 2020 and 3^rd^ March 2021. The carcasses were placed in open habitats (meadows, deadwood areas affected by bark beetle disturbance) and forests. At each deployment, two carcasses were placed in habitats with an open canopy (*n* = 22) and two others in relatively closed forests (*n* = 20), with one carcass placed on dry and the other on wet soil (dry: *n* = 21, wet: *n* = 21) (Figure 1). The setup used in carcass detection consisted of a DJI Matrice 200 V2 drone equipped with a Zenmuse XT2 thermal camera fitted with a FLIR Tau temperature sensor (longwave infrared thermal camera, Tau 2 thermal core, spectral range: 7.5–13.5 *μ*m, heat sensitivity <50 mK at f/1.0, 30** **Hz max. frame rates, 4 K visual sensor, 13 mm lens, and field of view: 25° × 19°) (https://www.dji.com/zenmuse-xt2, SZ DJI Technology Co., Shenzhen, China).

**Figure 2:**
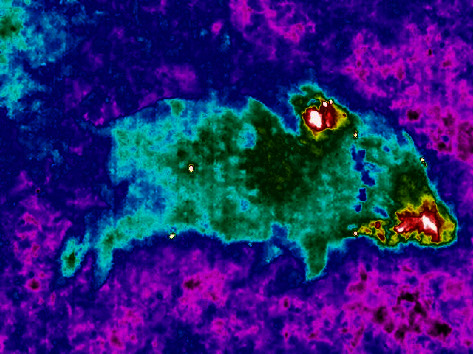
Thermal infrared image of a 10-day old wild boar carcass in autumn, showing the typical pattern of heat emission from the snout and behind the animal's neck, due to the first appearance of feeding fly maggots. Small white spots indicate insects on the carcass.

Flyovers took place directly from the nearest forest road in a straight line over the known carcass site, with a starting altitude above the carcass of 40 m. The drone was then navigated back and stopped directly over the location of the carcass. If the underlying vegetation allowed it, we descended the flight altitude to 25 m vertically towards the carcass. If a carcass was detected at 40 m, the flight altitude was raised vertically to 60 m to further evaluate the detection capability of the thermal camera. Using the HeatTrack function, the camera recognizes the hottest object in the camera's view and indicates it by a red point on the monitor of the remote controller, which provides a live feed of the thermal camera. Accordingly, a carcass was designated as found when the drone detected it as the warmest point, and if that point remained stable. If a point switched between the carcass and the surroundings, we did not consider this carcass as detected at that given flight altitude. At all flight heights, the carcass temperature (if measurable), air temperature, and coldest spot measured by the infrared camera of the drone were noted. The body temperature of each carcass was manually recorded based on measurements in the rectum, mouth, and on the carcass' surface and underside immediately before or after the drone flight, using a handheld thermometer (Voltcraft DT-300). The temperature of the maggot masses was also determined. Heat is not evenly distributed over the carcass, as it often develops first at natural body openings, where flies preferentially lay their eggs (Figure 2). As the thermal camera can detect local heat sources, the maximum measured temperature was used for the analysis. Temperature values were recorded based on stable values, i.e., those displayed within 10 s.

### 2.3. Environmental and Carcass Conditions

**Figure 3:**
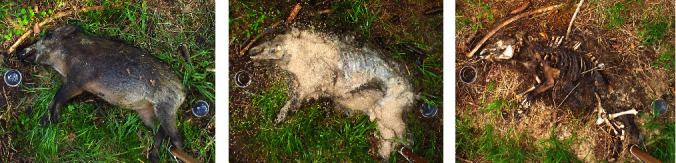
Typical decomposition of a wild boar carcass placed in a forest with wet soil and a closed canopy in summer 2020. The decomposition stage after deployment: (a) bloated (7 days); (b) post-bloated (14 days); (c) dry remains (42 days).

The temperature difference was calculated between the maximum temperature measured on or in the carcass using a handheld thermometer and the drone-based measurements. Habitat types were classified as meadow, deadwood area, mixed forest, coniferous forest, and deciduous forest based on Silveyra Gonzalez et al. [43]. The soil condition was either wet or dry, based on our observations. Canopy openness was calculated from hemispherical photographs taken with a 180° fisheye lens, and Gap Light Analyzer software [44] was then used to extract the percentages of openness (higher percentages represent greater canopy openness). Sky conditions were characterized as sunny or cloudy; dawn referred to flights that took place during nautical and civil dawn, i.e., in the early morning hours on a clear day with the sun still below the horizon. The air temperature was calculated as the mean of the air temperature measurements obtained by the thermal camera at the three flight altitudes: 25 m, 40 m, and 60 m. The number of flyovers for each identified carcass was also recorded. The decomposition stage was defined as fresh, putrefaction, bloated, post-bloated, advanced decay, and dry remains based on Lee Goff [46] and Anderson and Van Laerhoven [70] (Table 1 and Figure 3).

Further binary variables were the presence of maggot masses and the weight of the carcass. The difference between the maximum carcass temperature measured by a handheld thermometer and the air temperature was also calculated (Table 2).

### 2.4. Statistical Analysis

First, the accuracy of the carcass temperature measurements obtained by the thermal camera was assessed by comparison with the maximum carcass temperatures measured on the ground. For this, we fitted a generalized linear model regressing drone-based over handheld measurements. Measurement accuracy was evaluated using the coefficient of determination (*R*^2^) and root-mean-square error (RMSE, given in °C, range of the data from −1°C to 60°C). The variables that influenced the accuracy of the temperature measurements were determined in a generalized linear mixed model (GLMM) (model 1) within which the difference in the carcass temperature as measured with the thermal camera vs. manually was the dependent variable and flight altitude (25 m, 40 m, and 60 m), habitat type, canopy openness, air temperature, and sky conditions were the independent variables. A flight elevation of 40 m was defined as the reference, as most overflights were conducted at this elevation; flights at 25 m or 60 m elevation were not always possible. Canopy openness and air temperature were included using second-order orthogonal polynomials.

Two GLMMs with binomial distributions were then established to determine (model 2) which of the environmental parameters and (model 3) which of the carcass parameters influenced the detection probability of a wild boar carcass. Environmental variables were differentiated from carcass-specific factors because information on the former is available in advance to a drone pilot and allows the flight day or time to be adjusted accordingly, unlike carcass parameters, which are not known prior to carcass detection. Detection success served as the response variable for both models (1 = found; 0 = not found). The carcass ID number was included as a random intercept, and for model 2, the number of flights for each carcass was added as a random slope to account for differences in the number of flight days. Model 2 also included habitat type, soil condition, canopy openness, air temperature, and sky condition as independent variables (Table 2). Canopy openness and air temperature were included with a second-order orthogonal polynomial. In model 3, the explanatory variables were decomposition stage, presence of maggot masses, and the differences between the air temperature and the carcass temperature (second-order polynomial) (Table 2). The GLMMs were constructed using the R package glmmTMB [47].

Finally, two conditional inference trees (CTrees) were fitted to derive thresholds for the environmental and carcass-related factors that determine the probability of carcass detection, using the same variables as in models 2 and 3. CTrees are decision trees based on unbiased recursive partitioning using a significance test to select input variables. The package partykit [48] was used to build the CTrees [49]. A 27% proportion of observations was used to establish a terminal node; an increased minimum sum of weights in a terminal node was applied to calculate the influence of environmental variables; and a 46% proportion of observations was used to establish terminal nodes for possible predictor variables of the carcass.

All statistical analyses were performed using R 4.0.2 [50]. Statistical significance was assumed for *P* values <0.05.

## 3. Results

Carcasses were detected by thermal imaging during 145 of the 379 drone flights (38%). The detection rates (calculated as a percentage of the number of observations in each stage) for carcasses in different decomposition stages were fresh: 4 (14%), putrefaction: 10 (16%), bloated: 13 (36%), post-bloated: 26 (49%), advanced decay: 50 (47%), and dry remains: 42 (44%). The number (%) of carcasses detected according to habitat type was mixed forest: 56 (36%), coniferous forest: 24 (29%), deciduous forest: 8 (24%), deadwood area: 29 (38%), and meadow: 28 (97%). Flight speeds tested during 36 overflights with positive detection were measured with 9 m/s on average (median = 9.7 m/s, mean = 9.1 m/s, and max = 14 m/s).

**Figure 4:**
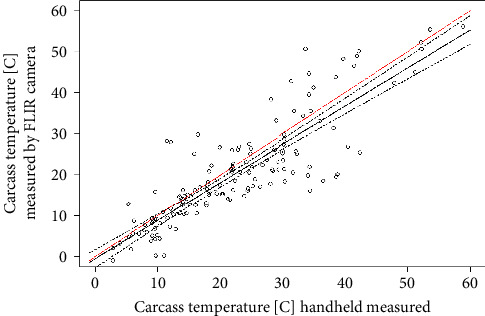
Temperature measurements by drone-based thermal imaging compared to measurements on the ground. *R*^2^ and RMSE in model 1 were 0.75 and 5.9 (°C) (within a data range of −1°C to 60°C), respectively. The red line represents a 1 : 1 line (indicating a perfect correlation).

In the test of the accuracy of the thermal camera, the generalized linear model yielded a value for *R*^2^ of 0.75 and the RMSE calculation value of 5.9°C (Figure 4). According to model 1, the accuracy of the thermal camera increased significantly and linearly with greater canopy openness (51.789 ± 8.382, *t* = −6.18, *P* < 0.001, orthogonal polynomial: coefficients estimated are not on the response scale) (Table 3). Sky conditions significantly increased measurement accuracy, with a higher accuracy obtained in flyovers conducted on days with an overcast sky (4.666 ± 1.269, *t* = 368, *P* < 0.001) or at dawn (8.337 ± 2.234, *t* = 3.73, *P* < 0.001). Model 1 also indicated a lower accuracy at a flight altitude of 60 m (1.242 ± 0.206, *t* = 6.02, *P* < 0.001) and a higher accuracy at a flight altitude of 25 m (−1.267 ± 0.206, *t* = −6.14, *P* < 0.001) than at a flight altitude of 40 m.

In model 2, the probability of carcass detection was significantly higher in meadows than in forests (3.920 ± 1.549, *t* = 2.53, *P*=0.011). The probability of carcass detection also increased significantly and linearly with greater canopy openness (28.331 ± 7.410, *t* = 3.82, *P* < 0.001) (Table 4) and was significantly higher on days with cloudy sky conditions than on days with sunshine (2.546 ± 0.486, *t* = 5.23, *P* < 0.001). In addition, on days with a clear sky, the carcass detection probability was higher in the early morning hours, when the sun was not yet above the horizon, than later in the day, under full sun (1.514 ± 0.677, *t* = 2.24, *P*=0.025). Also, the probability of carcass detection increased at warmer air temperatures and followed a curvilinear (second-order) relationship at higher temperatures (linear term: 15.530 ± 5.224, *t* = 2.97, *P*=0.003, quadratic term: −10.332 ± 3.991, *t* = −2.59, *P*=0.010).

**Figure 5:**
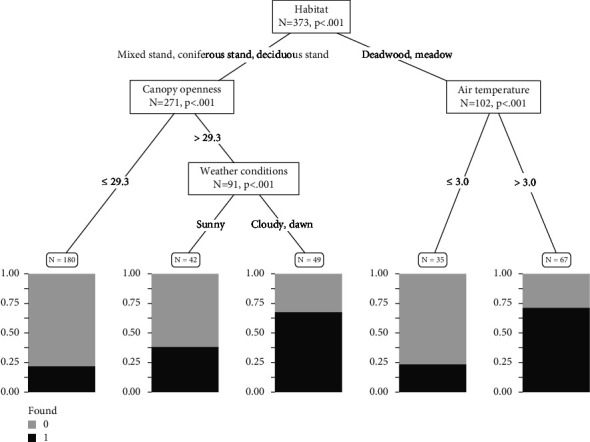
Conditional inference tree (CTree) predicting the influence of environmental factors on the probability of wild boar carcass detection using thermal imaging from drones. Each tree node represents a split of the data into significant subsets according to the contribution of variable importance. Split nodes include the number of observations and the significance level. The *x*-axis shows the predicted probability of carcass detection using drone-based thermal imaging (not found = 0, found = 1). Threshold values and factors responsible for splitting of the dataset are labeled on all branches.

Similar to model 2, the CTree for environmental variables provided significant thresholds (*P* < 0.01) for four variables: habitat, canopy openness, sky conditions, and air temperature (Figure 5). The first split of the decision tree indicated habitat as the main factor affecting the probability of carcass detection using thermal imaging, differentiating between forest types (mixed, coniferous, and deciduous) and open habitats (deadwood areas and meadows). Within forests, canopy openness was the next most relevant factor. According to our data, for a canopy openness of <29.3%, the probability of carcass detection was <25%. When the canopy openness exceeded 29.3% greater detection success was consistent with the influence of sky conditions, as flights conducted in cloudy weather or during early morning hours (dawn) resulted in a higher detection probability (<70%) than flights conducted in sunshine (<40%). The highest detection probability (>70%) was in open areas such as deadwood or meadows and at air temperatures >3.0°C; at colder temperatures, the detection probability was <25%.

Model 3 revealed a significant effect of the presence of maggot masses (1.444 ± 0.480, *t* = 3.01, *P*=0.003) (Table 5) and a significantly lower detection probability at the decomposition stage putrefaction than at the advanced decay stage (−1.328 ± 0.556, *t* = −2.38, *P*=0.017). A significant linear and quadratic relationship was determined for the difference between the air temperature and the carcass temperature (linear term: 15.246 ± 3.767, *t* = 4.05, *P* < 0.001, quadratic term: −10.987 ± 3.486, *t* = −3.15, *P*=0.002).

**Figure 6:**
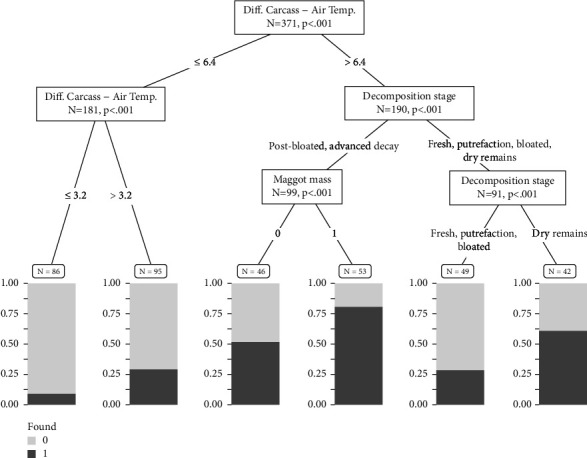
CTree used in the analysis of the influence of wild boar carcass factors on the probability of wild boar carcass detection using drone-based thermal imaging. For a detailed description of the CTree structure, see Figure 5.

Similar to model 3, the CTree provided significant thresholds (*P* < 0.01) for decomposition stage, presence of maggot masses, and the temperature difference between the carcass and the air. The latter was determined by the CTree algorithm as the most influential variable, with a threshold of 6.4°C (Figure 6). Temperature differences >6.4°C resulted in the highest probabilities (≤80%) of carcass detection, together with the presence of maggot masses in the decomposition stages post-bloated and advanced decay. The probability was still >60% for carcasses in the dry remains stage but <30% for those in the inner decay stages such as fresh, putrefaction, and bloated.

## 4. Discussion

This study assessed the potential advantages and limitations of drone-based infrared technology to locate wild boar carcasses and examined the factors that influence the detection probability. The rapid detection of carcasses is critical to the successful containment of emerging wildlife diseases such as ASF. Our results demonstrate that a drone equipped with a thermal camera can detect carcasses and accurately measure carcass temperatures. Greater canopy openness, cloudy sky conditions, and a lower flight altitude positively influenced the accuracy of the carcass temperature measurements, while open habitats such as meadows positively influenced the detection success. In forests, the detection probability was higher when canopy openness exceeded 30%, with higher success rates achieved during cloudy sky conditions or flights at dawn. An ambient temperature >3°C was shown to be necessary for carcasses to develop enough detectable heat. Detection success was also influenced by a difference between the carcass temperature and the ambient air temperature of >6.4°C. Higher success rates were possible for active carcass decay stages, especially post-bloating and advanced decay. These stages are characterized by the development of maggot masses, which was the most important determinant of detection success.

Our results revealed a fair match between the carcass temperature and the temperature measured by the thermal camera, which forms the basis of carcass detection. The observed accuracy was previously defined as the estimated average obtained from the small thermal sensors fitted on drones [51], and it corresponded with the accuracy of the Zenmuse XT2 thermal sensor, which is ±5°C (https://www.dji.com/zenmuse-xt2). The slight differences in accuracy can be attributed to surface emissivity and environmental factors such as air temperature, humidity, and sky conditions [52, 53]. These same factors might also explain the wider spread in the data at higher temperatures (Figure 4). Previous studies that tested the accuracy of thermal cameras reported slightly better values (e.g., *R*^2^ = 0.70–0.96 and RMSE = 3.18–5.45) because the tests were conducted either indoors in a temperature-controlled room or outside within controlled settings [54–57]. Cooled thermal cameras would be an alternative, as they provide greater sensitivity, higher spatial resolution, and faster frame rates, but they need to be carried by larger vehicles, such as helicopters, which are more costly and not readily available [53].

Our temperature measurements were influenced by flight altitude, as the accuracy decreased with increasing elevation. This can be attributed to atmospheric attenuation but also to the fact that a lower flight altitude allows the object of interest to fill more of the camera's field of view. The same dependency of flight height was reported in previous studies [27, 56, 58, 59]. However, Bodnar et al. [58] recommended flights at higher altitudes (50–100 m) because they allow the observation of a larger area at a time [58]. In the study of Lee et al. [28], carcasses were detected from a 1 km flight height using a thermal camera on a helicopter [28]. In our study, a 25 m flight altitude was not always possible because of tall trees, while at a 60 m flight altitude, both the measurement accuracy and the detection probability declined. Therefore, a flight height of 40 m offers a good compromise between the coverage of larger areas and a higher detection probability. Canopy cover also influenced the temperature measurements, with a denser canopy resulting in less accurate drone measurements. If a carcass was covered by vegetation, the thermal camera was not able to accurately measure its temperature, whereas for a carcass detected in habitats with less canopy cover, the temperature measurements were highly reliable. Within carcass temperatures ranging from −1°C up to 60°C, a RMSE of 5.89°C was deemed acceptable.

Our results showed that canopy openness, sky conditions, and habitat type influenced the detection probability of wild boar carcasses. The carcass detection probability was higher in meadows than in forests. Within the latter, the detection probability increased with greater canopy openness. A threshold of 29.3% openness was determined below which carcasses were unlikely to be found (detection probability <25%). This finding is in accordance with those of other studies in which the detection of wildlife in different forest types became increasingly limited with denser canopy cover [34, 60]. Therefore, drone-based searching should preferably be conducted in more open areas, given the very high probability of missing carcasses in densely forested areas. This limitation is a drawback of drone use in efforts to prevent or reduce the spread of ASF, as infested wild boars are often found in continuous forests [61] and typically withdraw to denser, mostly young forests, for their deathbeds [7, 22, 24]. In denser forests, a flight design with overlapping overflights might increase the detection probability. Furthermore, carcass searches with specially trained dogs might help to increase the detection rate. Several studies have tested the ability of this approach in the search for remains under different ecological conditions and were able to show that, in the detection of carcasses, dogs were more efficient, with a higher detection rate and a shorter time span between detections, than humans alone [62–64]. However, whether the use of dogs to detect wild boar carcasses will have a sufficient impact on the containment of ASF still needs to be tested.

Sky conditions also influenced the detection probability. Flyovers were successful when the sky was cloudy, as the lack of sunshine prevented heating of the surrounding vegetation. Based on our experience, the latter can happen within minutes after direct sunlight exposure. Especially treetops or tree stumps can heat up quickly. Thermal cameras do not differentiate between emitted or reflected heat [38], such that reflected heat is detected with a similar signature as the heat emitted from a carcass [39]. Masking effects due to sunshine and reflected solar radiation are a well-known problem in the application of thermal cameras in ecology [27, 28, 38, 39]. Direct solar radiation can also raise the temperature of a carcass, by 10–20°C compared to a carcass in the shade [39]. Within our study, the thermal camera measured carcass temperatures up to 65.5°C, which were likely reached by the absorption of direct sunlight (open canopy, ambient temperature ≈ 30°C). We therefore recommend that, in searching for carcasses using thermal imaging, the searches should be conducted on overcast days or in the early morning hours of clear days, when the sun is still below the horizon and does not heat up the carcass or the surrounding vegetation.

Ambient air temperature also significantly affected the carcass detection probability. The warmer the air temperature during the flyover, the greater the likelihood of carcass detection. Our analysis showed that the probability of carcass detection was <25% when the ambient air temperature was <3°C. At these lower temperatures, the slow rate of metabolic decomposition and the absence of insects [65] hinder the rise in carcass temperature. During winter, carcass decomposition is much slower [59]. In the study of Hohmann et al. [39], at temperatures <10°C, there were no signs of carcass decomposition or heat development. Based on our observations, carcass detection with thermal cameras on colder days was only possible if the carcass was already in an advanced stage of decomposition. At this stage, maggots had most likely already invaded the carcass during warmer temperatures while at colder temperatures microbial activity continued to emit heat. By contrast, wild boars that die in late autumn or winter often decompose very slowly, with no noticeable signs of decomposition for several weeks or months [66]. Therefore, the applicability of thermal imaging to detect wild boar carcasses in winter is rather limited.

Our analysis of carcass parameters identified the variable temperature difference between the carcass and the ambient temperature as the most important, with a threshold difference of 6.4°C needed for detection. This result is in accordance with those of Lee et al. [28], who determined that a threshold of ≥8°C is sufficient for carcass detection by thermal imaging [28]. A relatively large temperature difference was previously shown to be crucial for the use of thermal devices in carcass location [59]. The presence of maggot masses also significantly increased the detection probability. When maggot masses were present on a carcass, our analysis revealed with up to 80% the highest chance for detection (Figure 6). This is in accordance with results from Butters et al. [27], who found that heat detection peaked with insect activity [27] while Lee et al. [28] showed that, in the absence of maggots, the detection probability decreased [28]. Maggot masses develop in larger amounts when the air temperature is warmer [67] and emit proportionally more heat [68]. Temperatures in the range of 22–25°C favor the development of maggot masses [26], which in turn can cause a carcass to become much warmer than the surrounding air. On overcast days, the largest differences recorded between the manually determined carcass temperature and air temperature and between the carcass temperature measured by the thermal camera and the air temperature were 36.3°C and 17.9°C, respectively (carcass with maggots present, advanced decay, 62 days post-mortem, air temperature: −1.7°C, max. carcass temperature = 34.5°C, 04.05.2021, 07:50). During flyovers on sunny days, the largest difference between the carcass temperature as recorded by the thermal camera and the air temperature was 31°C. Studies measuring ground-based carcass temperatures reported that these peak at ∼26°C above air temperature [29, 39]. In comparisons of carcass temperature measured by thermal cameras and the surrounding ground area temperature, the differences were in the range of ∼8–12°C [27, 28]. Amendt et al. [29] recorded a maximum carcass temperature of ∼40°C, reached in the presence of maggot masses. In our study, in the presence of maggot masses and relatively large temperature differences, carcass detection was at times possible even in forests with increased canopy closure.

In the usual process of decomposition, fly maggots develop on a carcass during active decay stages and form masses mostly during the post-bloating stage, which enables further the advanced decay stage [69, 70]. In our study, carcasses in the fresh and putrefaction stages of decomposition had a lower detection probability (<30%, 4 out of 28) than those in the advanced decay stage. During the stages of active decay, when maggots are present, the detection probability was ≥75%. Fresh refers to the first stage after death, with cooling of the body, until the first signs of inner putrefaction appear [70]. Following the death of an animal, the body cools quickly [29] such that the probability of detection of a still-warm body by thermal imaging is possible only within a very limited period. Most of the carcasses detected in our successful flyovers were in the advanced decay stage, which was for our study also the longest decomposition stage. Although, during this stage, maggots start to abandon the carcass in order to pupate, the residual emitted heat is usually sufficient to be detected by a thermal camera. Lee et al. [28] observed similar detectability and highlighted the usage of thermal imaging for searching purposes in the advanced decay stage [28]. The detection rate in carcass searches will be even higher when flyovers are conducted during a period of warmer ambient temperatures, when the likelihood of the presence of maggot masses is also higher [28, 59].

Our analysis revealed a probability of over 60% to detect carcasses in the stage of dry remains. There is evidence that microbially mediated decomposition can raise carcass temperatures even in the absence of maggots or solar radiation [30, 58]. Accordingly, the successful detection in this study of carcasses in the dry remains stage (detectable even at colder temperatures) can therefore most likely be explained by microbial processes. Des Marais [59] tested thermal imaging on pig carcasses and found no difference in the temperature of carcasses in the dry remains stage vs. that of the surroundings [59], whereas in the study of Hohmann et al. [39], carcasses were detected as late as after day 50 [39]. Amendt et al. [29] and Lee et al. [28] also detected carcasses several weeks post-mortem, but those still showed the presence of maggots or insect activity and were in the advanced decay stage [28, 29]. The detectability of older carcasses with thermal imaging may be useful in the management of ASF [28], especially during colder seasons, as older carcasses in late stages of decomposition may still be an active source of ASF infection. It has been shown that ASFV remains infectious at colder temperatures for several months, and that wild boars have contact with dead conspecifics weeks after death [7, 10, 14]. In general, wild boars seem to approach carcass sites containing carcasses of their conspecifics on average after a week, with direct contact after around 2 weeks and often in later stages of decomposition [71]. Therefore, the detection of older carcasses can contribute to the successful management of ASF and other diseases.

## 5. Conclusion

Using drones for the rapid detection of wild boar carcasses to prevent the spread of diseases, in particular ASF, is gaining increasing interest. This study demonstrated the feasibility of drone-based thermal imaging to detect large-animal carcasses, but the success of this approach depends on environmental and carcass factors. Our results provide guidelines for the optimization of search efforts, by identifying the conditions leading to the highest probability of detection: cloudy days, early morning hours, ambient temperatures >3.0°C, and canopy openness >30%. Under these conditions, the benefits of drones, i.e., their rapid coverage of large areas, including early succession stages of deadwood areas with difficult access, can best be exploited. In other habitats and conditions, complementary methods, such as the use of dogs, will still be needed. Further research should examine whether the search for infected carcasses can be improved using thermal cameras with higher sensitivities during more favorable conditions, for example, at night. It should also be systematically tested up to which flight speeds thermal cameras can detect carcasses. The fundamental knowledge presented herein will contribute to the development of measures aimed at preventing the spread of wildlife diseases, such as ASF in wild boar.

## Figures and Tables

**Table 1:** The six decomposition stages used in the statistical analysis, based on Anderson and Van Laerhoven [45] and Lee Goff [46].

Decomposition stage	Distinguishing features
Fresh	First post-mortem stage, starting with cooling of the body temperature after death and chemical breakdown of the body
Putrefaction	Digestion of body tissue by anaerobic bacteria
Bloated	Visible inflation of the abdomen due to gas production
Post-bloated	Skin breakage due to gas accumulation and insect or maggot activity, deflation of the body, removal of most of the body's flesh by large maggot masses
Advanced decay	Carcass left by the maggots for pupation, continued removal of the remaining flesh by Coleoptera and other arthropods
Dry remains	Only bones and skin with fur remaining

**Table 2:** Characteristics of the variables used in the statistical analysis.

Variable	Units/factors	Model 1	Model 2	Model 3
		Accuracy of the aerial thermal camera	Detection success—environmental conditions	Detection success—carcass conditions
Carcass number	ID number	*X*	*X*	*X*
Flight days	Number of flight days for each carcass		*X*	
Habitat type	Meadow, deadwood (bark beetle area), deciduous forest, coniferous forest, mixed stand	*X*	*X*	
Canopy openness	%	Quadratic	Quadratic	
Soil condition	Wet, dry		*X*	
Sky conditions	Cloudy, sunny, dawn	*X*	*X*	
Air temperature	°C	Quadratic	Quadratic	
Carcass temperature (handheld) vs. air temperature	°C			Quadratic
Decomposition stage	Fresh, putrefaction, bloated, post-bloated, advanced decay, dry remains			*X*
Maggot mass	Presence, absence			*X*
Carcass weight	kg			*X*
Flight altitude (m)	25, 40, 60	*X*		

**Table 3:** Estimated regression parameters, standard errors, *t* values, and *P* values for generalized linear mixed-effect model 1, evaluating the effect of environmental variables and different flight altitudes on the accuracy of temperature measurements of the drone-based thermal camera.

	Estimate	SE	*t* value	*P* value
Intercept	−1.872	1.151	−1.627	0.104
Habitat: coniferous stand	−1.068	1.424	−0.750	0.453
Habitat: deadwood	1.661	1.432	1.160	0.246
Habitat: deciduous stand	1.214	2.780	0.437	0.662
Habitat: meadow	0.750	1.447	0.518	0.604
Canopy	−51.789	8.382	−6.178	<0.001
Canopy (polynomial)	6.273	8.611	0.728	0.466
Air temperature	11.157	10.328	1.080	0.280
Air temperature (polynomial)	5.075	8.595	0.590	0.555
Sky conditions: cloudy	4.666	1.269	3.677	<0.001
Sky conditions: dawn	8.337	2.234	3.730	<0.001
Flight altitude 25 m	−1.267	0.206	−6.140	<0.001
Flight altitude 60 m	1.242	0.206	6.019	<0.001

Air temperature was included using a second-order orthogonal polynomial.

**Table 4:** Parameter estimates of generalized linear mixed-effect model 2, assessing the influence of environmental parameters on the probability of wild boar carcass detection using drone-based thermal imaging.

	Estimate	SE	*t* value	*P* value
Intercept	−2.020	0.724	−2.790	0.005
Habitat: coniferous stand	0.186	0.758	0.245	0.806
Habitat: deadwood	−0.272	1.334	−0.204	0.838
Habitat: deciduous stand	−0.489	0.984	−0.496	0.620
Habitat: meadow	3.921	1.549	2.531	0.011
Soil condition: wet	−0.344	0.691	−0.498	0.618
Canopy openness	28.331	7.412	3.823	<0.001
Canopy openness (polynomial)	−1.192	3.368	−0.187	0.851
Sky conditions: cloudy	2.546	0.487	5.233	<0.001
Sky conditions: dawn	1.515	0.678	2.235	0.025
Air temperature	15.530	5.224	2.973	0.003
Air temperature (polynomial)	−10.332	3.991	−2.589	0.010

Canopy openness and air temperature were included using second-order orthogonal polynomials.

**Table 5:** Estimated regression parameters, standard errors, *t* values, and *P* values for generalized linear mixed-effect model 3, evaluating the effect of wild boar carcass factors on the probability of wild boar carcass detection using drone-based thermal imaging.

	Estimate	SE	*t* value	*P* value
Intercept	0.267	0.862	0.310	0.757
Decomposition stage: fresh	−0.608	0.759	−0.801	0.423
Decomposition stage: putrefaction	−1.328	0.556	−2.388	0.017
Decomposition stage: bloated	−0.582	0.594	−0.981	0.288
Decomposition stage: post-bloated	−0.414	0.513	−0.804	0.402
Decomposition stage: dry remains	−0.264	0.469	−0.561	0.554
Maggot mass presence	1.444	0.480	3.009	0.003
Weight	−0.019	0.017	−1.118	0.267
Difference in cadaver temperature vs. air temperature	15.246	3.767	4.046	<0.001
Difference in cadaver temperature vs. air temperature (polynomial)	−10.987	3.486	−3.152	0.002

## Data Availability

The data used to support the findings of this study are available from the corresponding author upon reasonable request.
